# Silent Human* Trypanosoma brucei gambiense* Infections around the Old Gboko Sleeping Sickness Focus in Nigeria

**DOI:** 10.1155/2016/2656121

**Published:** 2016-01-31

**Authors:** Karshima Solomon Ngutor, Lawal A. Idris, Okubanjo Oluseyi Oluyinka

**Affiliations:** ^1^Department of Animal Health, Federal College of Animal Health and Production Technology, PMB 001, Vom, Nigeria; ^2^Department of Veterinary Parasitology and Entomology, Ahmadu Bello University, PMB 1045, Zaria, Nigeria

## Abstract

*Trypanosoma brucei gambiense* causes Gambian trypanosomosis, a disease ravaging affected rural parts of Sub-Saharan Africa. We screened 1200 human blood samples for* T. b. gambiense* using the card agglutination test for trypanosomosis, characterized trypanosome isolates with* Trypanosoma gambiense* serum glycoprotein-PCR (TgsGP-PCR), and analyzed our data using Chi square and odds ratio at 95% confidence interval for statistical association. Of the 1200 samples, the CATT revealed an overall infection rate of 1.8% which ranged between 0.0% and 3.5% across study sites. Age and sex based infection rates ranged between 1.2% and 2.3%. We isolated 7 (33.3%) trypanosomes from the 21 seropositive samples using immunosuppressed mice which were identified as* T. b. gambiense* group 1 by TgsGP-PCR. Based on study sites, PCR revealed an overall infection rate of 0.6% which ranged between 0.0% and 1.5%. Females and males revealed PCR based infection rates of 0.3% and 0.8%, respectively. Infection rates in adults (1.3%) and children (0.1%) varied significantly (*p* < 0.05). We observed silent* T. b. gambiense* infections among residents of this focus. Risks of disease development into the second fatal stage in these patients who may also serve as reservoirs of infection in the focus exist.

## 1. Introduction 

*Trypanosoma brucei gambiense* causes the Gambian sleeping sickness, a very chronic, debilitating, complex, and fatal parasitic zoonosis ravaging affected rural parts of Sub-Saharan Africa. The disease transmitted by tsetse flies is widespread in the Sub-Saharan African region posing serious public health problems in the region and to tourists visiting tropical Africa [1, 2]. It is a prototype of a neglected zoonotic pathogen in terms of drug development and sustainable control programmes.

In natural conditions, transmission of the parasite is cyclical through bites of infected tsetse flies including* Glossina palpalis, G. tachinoides*, and* G. fuscipes*. These vectors are especially common at watering places like rivers or lakes where people frequently visit to collect water and do their washing and animals visit to drink water [3]. The parasite is divided into two subtypes; type 1 causes a more chronic disease and represents about 90% of all cases of Gambian trypanosomosis, while type 2 is said to be associated with an acute like disease and represents the remaining 10% of the disease [4, 5].

The pathogen is ranked 9th of the 25 human infectious diseases in Africa based on its socioeconomic impact [6] and is incriminated in over 15,000 new cases yearly with the majority of these cases ending fatally [7]. Seventy million people are continuously exposed to the risk of infection in 38 Sub-Saharan African countries where active transmission is reported and only 5–7% of the population at risk is covered by surveillance [7]. Some of these infections may be latent and may remain unnoticed until they get to the second fatal stage [8].

In Nigeria however, human infection with* T. b. gambiense* in the Gboko sleeping sickness focus was first reported in 1974, and since then no successful attempt has been made to control the disease in the affected region [9]. Considering the fact that this infection is still not yet routinely diagnosed in Nigerian hospitals even in endemic areas and the risk associated with parasites invasion of the central nervous system and producing fatal disease, we designed this study to conduct an active screening of* T. b. gambiense* in humans in the old Gboko sleeping sickness focus in Nigeria and characterized isolates using TgsGP-polymerase chain reaction.

## 2. Materials and Methods

### 2.1. Study Area

**Figure 1:**
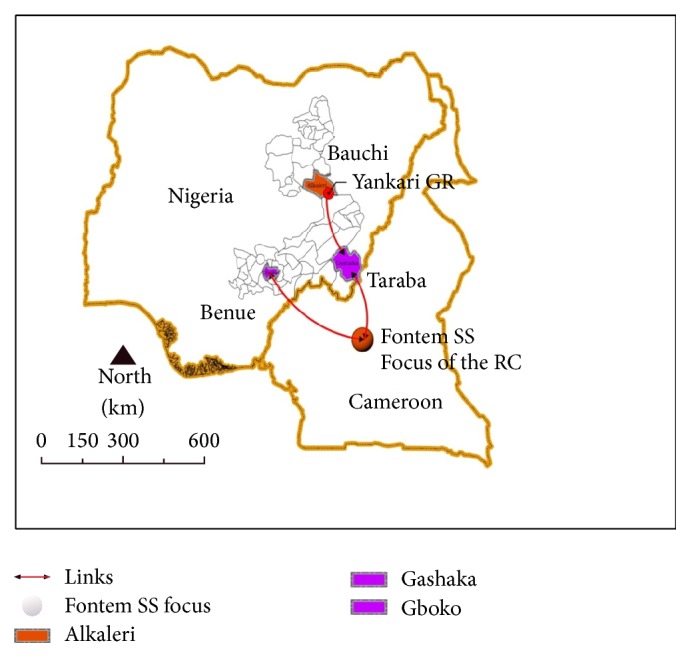
Links between the study sites and possible sources of* T. b. gambiense* infections. GR: game reserve; RC: Republic of Cameroon; SS: sleeping sickness.

This study was carried out around the old Gboko sleeping sickness focus which is located in the northern part of Nigeria between longitudes 7°47′ and 10°00′ east and latitudes 60°25′ and 8°8′ north. It shares boundaries with five other states, namely, Nasarawa (north), Taraba (east), Cross River (south), Enugu (southwest), and Kogi (west), and with the Republic of Cameroon to the southeast (Figure 1). The major occupation in this region is agriculture, particularly crop and livestock farming, as well as fishing.

### 2.2. Study Design

We conducted a cross-sectional study around the old Gboko sleeping sickness focus in Nigeria within six Local Government Areas (LGAs), namely, Gashaka, Gboko, Ibi, Karim Lamido, Ukum, and Vandeikya, considering their relationships with the Gboko and Fontem sleeping sickness foci and the Gashaka-Gumti and Yankari game reserves. Human subjects were sampled systematically from each household by selecting every 5th household following the order in which houses were built, and individuals were selected using balloting by names.

### 2.3. Sample Collection

#### 2.3.1. Blood Sampling of Human Subjects

Two millilitres of blood was aseptically collected from each human subject via the median cubital vein using a 5 mL syringe and 21 G needle and transferred immediately into clean labelled sample bottles containing ethylene diamine tetra-acetic acid (EDTA) at 1.5 mg/mL of blood [10] and gently shaken until the blood was properly mixed with the anticoagulant and analyzed within 1-2 hours after collection [11]. These samples were subjected to the card agglutination test for* T. b. gambiense* as described by Magnus et al. [12]. All CATT positive samples were then inoculated into mice immunosuppressed with cyclophosphamide to isolate trypanosomes.

### 2.4. Laboratory Analysis of Samples

#### 2.4.1. The Card Agglutination Test for Trypanosomosis (CATT)

The CATT test kits were obtained from the Institute for Tropical Medicine, Antwerp, Belgium. Blood samples were analyzed using the CATT as described by Magnus et al. [12]. This test is based on the detection of* T. b. gambiense* specific LiTat 1.3 antibodies using a purified* T. b. gambiense* variable surface antigen.

#### 2.4.2. Mice Infection and Parasitaemia Estimation

Mice were obtained from the Small Animal Experimental Unit of the National Veterinary Research Institute, Vom, Nigeria, for the study. The mice were appropriately labelled and screened for ectoparasites and endoparasites by standard techniques [13] and acclimatized for two weeks before inoculation. The mice were immunosuppressed using intraperitoneal administration of cyclophosphamide at 200 mg per kg body weight. About 0.5 mL of each seropositive blood sample was enriched with 0.5 mL of phosphate saline glucose (PSG) buffer and 0.3 mL of the PSG buffer enriched seropositive blood from each seropositive sample was then inoculated into an intraperitoneally immunosuppressed mouse. Tail-blood samples were collected from each mouse every 24 hours to estimate parasitaemia using the rapid matching technique as described by Herbert and Lumsden [14].

#### 2.4.3. Isolation and Purification of Trypanosomes

The bloodstream form trypanosomes isolated from humans and propagated in mice were separated from the infected mice blood using a DEAE 52 column (Whatman, Maidstone, Kent, UK) as described by Lanham and Godfrey [15]. These parasites were then stored at 4°C until needed for DNA extraction.

#### 2.4.4. Extraction of DNA from Purified Trypanosomes

Trypanosome DNA extraction was done using GeneJET genomic DNA extraction kit (Thermo Scientific, Germany) using the method described by Oury et al. [16]. The extracted DNA was stored at −20°C until needed for PCR at the Biotechnology Laboratory of the Ahmadu Bello University, Zaria, Nigeria.

#### 2.4.5. Detection of* Trypanosoma gambiense* Specific Glycoprotein (TgsGP)

The TgsGP primer set with sequences forward 5′-GCTGCTGTGTTCGGAGAGC-3′ and reverse 5′-GCCATCGTGCTTGCCGCTC-3′ [17] was used to characterize the* Trypanosoma* isolates. Cycling conditions for TgsGP-PCR were denaturation at 98°C for 10 seconds to activate the Phusion Flash II DNA Polymerase, followed by 40 cycles with denaturation at 98°C for 1 second, annealing at 63°C for 30 seconds, 30-second elongation at 72°C, and a final extension at 72°C for 5 minutes as recommended by the manufacturer. All amplified products were analyzed by electrophoresis in a 2% agarose gel and UV illumination after ethidium bromide staining.

### 2.5. Data Analysis

All data obtained during the study were analyzed using GraphPad Prism 4.0. Infection rates of* T. b. gambiense* were calculated by dividing the number of infected individuals by the total number of individuals examined and expressed as percentages. This was done for different variables such as study sites, sex, and age. The Chi square (*χ* ^2^) test and odds ratio were used where appropriate to compare the prevalence rates based on different variables and values of *p* < 0.05 were considered significant.

## 3. Results

A total of 1200 human blood samples from 6 sites around the old Gboko sleeping sickness focus were analyzed using the CATT and PCR for the presence of* Trypanosoma brucei gambiense* as shown in Table 1.

The CATT revealed an overall infection rate of 1.8% of the 1200 samples studied. Based on the 6 sites studied, Gashaka, Gboko, Ibi, Karim Lamido, Ukum, and Vandeikya recorded infection rates of 3.5% (7/200), 2.5% (5/200), 0.5% (1/200), 2.5% (5/200), 0.0% (0/200), and 1.5% (3/200), respectively (Table 1). Sex based infection rates were 1.2% (7/600) and 2.3% (14/600) for females and males, respectively (Table 2). Infection rates recorded by adults and children were 2.2% (7/200) and 1.5% (7/200), respectively (Table 3).

Trypanosomes were isolated from 7 (33.3%) of the 21 seropositive samples using immunosuppressed mice. These isolates were characterized using the* Trypanosoma gambiense* serum glycoprotein- (TgsGP-) PCR as group 1 of* T. b. gambiense.* PCR revealed an overall infection rate of 0.6% of the 1200 samples analyzed. PCR based infection rates in relation to study sites were 1.5% (3/200) and 1.0% (2/200) for Gashaka and Gboko, respectively (Table 1). Both Karim Lamido and Vandeikya recorded 0.5% (1/200) while 0% (0/200) infection rates were recorded by both Ibi and Ukum (Table 1). Females and males revealed infection rates of 0.3% (2/600) and 0.8% (5/600) while there was significant variation (*p* < 0.05) between the 1.3% (6/450) and 0.1% (1/750) infection rates recorded by adults and males, respectively.

## 4. Discussion

In our study, we inoculated human seropositive samples into mice to propagate* T. b. gambiense* considering the low parasitaemia associated with this parasite. Mice were inoculated with cyclophosphamide at 200 mg per kg body weight to suppress mice immunity due to the low parasite isolation rate reported for this parasite [18]. Isolated trypanosomes were also purified to ensure that DNA from mice blood does not contaminate trypanosome DNA which was required for polymerase chain reaction.

We observed silent* T. b. gambiense* infections in this old sleeping sickness focus. Previous report showed that Gboko was endemic for the pathogen [9]. The findings of our study 4 decades after the first report in the region still indicate that the area may still be endemic for HAT and tsetse probably due to lack of or inadequate stakeholders' efforts towards the control and eradication of the disease. This region shares border with the Fontem sleeping sickness focus of the Republic of Cameroon, thus making transboundary transmission a possibility. The riverine nature of this region makes it suitable for tsetse habitat [3] and therefore is a probable reason for the occurrence of this infection. Other factors which include availability of several wildlife species in the Gashaka-Gumti and neighbouring Yankari game reserves and the occupational hazards through farming and fishing [19] might have contributed to the occurrence of the disease. Furthermore, evidence of lack of sustained vector control measures due to lack of funds [20], the high cost and unavailability of trypanocides used against* T. b. gambiense*, and the increasing trend of treatment failure [21] might have also contributed to the occurrence of this infection in the region.

The overall seroprevalence rate of 1.8% revealed by this study is lower than the earlier report by Karshima et al. [22] in a region of Taraba State. The higher prevalence observed in Gashaka may be attributable to the presence of the Gashaka-Gumti National Park which harbours several wildlife species that can serve as reservoirs of* T. b. gambiense*. The extension of the Yankari game reserve forest into the Jevjev and Binari areas of Karim Lamido may explain also the high prevalence observed in the area. The riverine nature of this region might have contributed to the high prevalence observed as riverine areas are known to promote tsetse breeding.

The variations in the prevalence of* T. b. gambiense* in relation to sex are in agreement with the earlier report by Karshima et al. [22], who also reported higher prevalence in males than in females in a region of Taraba State. This may be due to the greater involvement of males in occupations such as farming, fishing, and hunting which put them at more risk than the females. This finding however contradicts the report of Mohammed et al. [23] who reported higher prevalence in females in Southern Sudan probably due to differences in vegetation, tsetse density, and vectorial capacities of tsetse flies in the Sudan. Although there was no significant difference in the seroprevalence in adult and children, the prevalence in children may not be unconnected with their practice of swimming around rivers and streams which may expose them to tsetse bites.

One of the major objectives of this study was to isolate and characterize trypanosome isolates from serologically positive humans. This objective was achieved by the detection of the TgsGP gene which is present in only one strain of the 3 trypanosomes pathogenic to man. This gene is specific to group 1* T. b. gambiense* and is shown to confer resistance to human serum. We were able to isolate and characterize 7 stocks from 21 CATT seropositive humans. The high specificity of the PCR technique may be a reason for the low infection rate revealed by the technique. On the other hand it was also possible that the CATT detected inactive infections where antibodies have not completely disappeared from the blood.

The epidemiological and clinical significance of detecting only one species of human infective trypanosomes in the study area is that the risk of treatment failure associated with mixed infections of* T. b. rhodesiense* and types 1 and 2* T. b. gambiense* which respond differently to HAT chemotherapy [24] will be overcome. It was also not surprising to have detected only type 1* T. b. gambiense* among all human isolates since it has been reported to be associated with over 90% of cases of human African trypanosomosis [4, 5].

In conclusion, we observed silent* T. b. gambiense* infections among residents of this old sleeping sickness focus. Infections were more prevalent in children and males. The epidemiological implication of this finding is the risk of tsetse flies spreading these silent infections to uninfected population.

## Figures and Tables

**Table 1:** PCR and CATT based prevalence of *Trypanosoma brucei gambiense* in relation to study sites.

Study sites	Number examined	CATT positive	TgsGP-PCR positive
Gashaka	200	7 (3.5)	3 (1.5)
Gboko	200	5 (2.5)	2 (1.0)
Ibi	200	1 (0.5)	0 (0.0)
Karim Lamido	200	5 (2.5)	1 (0.5)
Ukum	200	0 (0.0)	0 (0.0)
Vandeikya	200	3 (1.5)	1 (0.5)
Total	**1200**	**21 (1.8)**	**7 (0.6)**
*χ* ^2^	—	**10.32**	**5.892**
*p* value	—	**0.066**	**0.3169**

**Table 2:** PCR and CATT based infection rates of *Trypanosoma brucei gambiense* in relation to sex.

Sex	Number examined	CATT positive	TgsGP-PCR positive
Female	600	7 (1.2)	2 (0.3)
Male	600	14 (2.3)	5 (0.8)
Total	**1200**	**21 (1.8)**	**7 (0.6)**
*χ* ^2^	—	**2.375**	**1.293**
*p* value	—	**0.1233**	**0.2554**
Odds ratio	—	**0.4941**	**0.3980**

**Table 3:** PCR and CATT based infection rates of *Trypanosoma brucei gambiense* in relation to age.

Age	Number examined	CATT positive	TgsGP-PCR positive
Adult (≥18 years)	450	10 (2.2)	6 (1.3)
Children (≤17)	750	11 (1.5)	1 (0.1)
Total	**1200**	**21 (1.8)**	**7 (0.6)**
*χ* ^2^	—	**0.9338**	**6.946**
*p* value	—	**0.3339**	**0.0082**
Odds ratio	—	**1.527**	**10.08**
